# First District Dental Society of New York

**Published:** 1887-01

**Authors:** 


### FIRST DISTRICT DENTAL SOCIETY OF NEW YORK.

REPORTED EXPRESSLY FOR THE INDEPENDENT PRACTITIONER.

The society held a regular meeting at the rooms of the S. S. White Dental Manufacturing Co., corner of Broadway and 32d Street, on Tuesday, November 2d, at eight o'clock, the President, Dr. Wm. Carr, in the chair. After the minutes of the last meeting were read, and several new members had been elected (one of whom was Dr. Wm. Herbst, of Bremen, elected an honorary member), the President called for the report of the Clinic Committee, which was read by the chairman, Dr. Bödecker, who reported an interesting clinic, with an attendance of about one hundred and ten persons. Dr. M. L. Rhein filled, by the Herbst method, cavities in the proximate and grinding surfaces of a first and second superior bicuspid. In the mouth of the same patient were some other large fillings, inserted in a clinic about three years ago, which were in good condition.

Dr. J. P. Geran, of Brooklyn, also filled a right lower first molar by the Herbst method, the cavity involving the mesial and grinding surface.

Dr. C. S. W. Baldwin demonstrated and explained the setting of a Logan crown, with gold cap over the root.

Dr. Lœwenberg filled, in seven minutes, a left lower first molar with Steuver's new gold, the cavity occupying the grinding surface of the tooth.

Dr. C. P. Brown exhibited some of his porcelain crowns with the new pins, which are placed in the crown in such a manner as not to be in the way of close articulations on the lingual surface, or to shine through the porcelain of the labial surface.

Dr. B. Hess presented a cabinet containing many interesting abnormalities in extracted teeth.

Dr. O. F. Coe exhibited the model of a case of irregularity, and an extracted tooth with an osteoma.

Dr. Geo. A. Mills showed a set of universal hand pluggers, with serrations upon the sides as well as the points of the instruments, to be employed for lateral as well as direct pressure.

Dr. C. F. W. Bödecker filled an extracted molar tooth with gold, by the Herbst method.

Dr. J. G. Morey presented a patient with necrosis of the lower jaw, in the region of the right lower first molar.

Dr. L. B. Wilson sent a letter regretting his inability to attend the clinic, but forwarded another sample of his plastic-gold white alloy for front teeth, and some specimens of pivot teeth. Dr. Bödecker did not desire to take the responsibility of the trial with the new gold upon himself, and proposed to divide it between Drs. Wm. H. Atkinson, Wm. H. Dwinelle and Wm. Carr, who would give the result of their trials at the next meeting.

Dr. Bödecker then announced that he could no longer answer all the letters of inquiry sent to him by other dental practitioners, as the number he had received last month amounted to thirty-six. He would be glad to give any verbal information in regard to cases, etc., at the clinic, or even at his office, upon appointment.

Dr. Wm. H. Dwinelle made a partial report upon the plastic gold tried at the last clinic. He was sorry to state that he had not been able to continue the experiment to-day, as he had been out of the city, but would exhibit it at the next clinic. As far as he had gone, he liked Dr. Steuver's gold very much, but he was wedded to his old love, Morgan's crystal gold. Dr. L. B. Wilson's gold he believed to be impure, or at least coated with shellac or some other resinous varnish, thereby enabling it to be packed by a heated instrument, but he was sure that every sensible dental practitioner knew that such a filling would not stand the test of time. He was, however, willing to give it another trial.

The President then called for the reports of special committees, when Dr. C. F. W. Bödecker made the report on the Herbst reception and clinics, all of which have been published in this journal.

Dr. D. W. Tenison reported that a special clinic was held at the rooms of the S. S. White Dental Manufacturing Co., on Friday, October 15th, when Dr. W. J. Younger was prepared to implant teeth into artificial sockets. There were two patients present, but in one case the space in which the tooth was to be implanted had been partially obliterated by the adjacent teeth, and in the other the patient was too timid to submit to the operation. Dr. Charles L. Andrews, however, volunteered to submit to an operation, and Dr. Younger extracted a devitalized lower central incisor, which stood so irregularly that the operator was obliged to make a new socket before a substitute could be implanted. Dr. Andrews has suffered no inconvenience since the operation, which, so far, seems to be successful. A very peculiar phenomenon which seems to attend these operations, is that teeth which, when first implanted, are not of the same color, in a short time seem to take on the hue of their near neighbors.

Dr. W. W. Walker announced that the President would call for a special meeting of this society, to be held Friday, November 19th, at eight o'clock P. M., when Dr. N. W. Kingsley would read a paper entitled "Dentistry Not a Specialty of Medicine." (This paper will be found on page 1 of the present number.—EDITOR.)

The President called for incidents of office practice. Dr. Vanderpant exhibited two models, with supernumerary teeth bearing distinct marks of hereditary syphilis.

Dr. W. D. Tenison reported the case of a lady who had worn an artificial plate bearing a right upper central incisor for over fifteen years, and which had been a great annoyance during all that time. On Dr. Younger's arrival in New York, he implanted a tooth in the space. There was a slight swelling around it the next morning, but this soon abated, and at present it is quite firm and the surroundings seem healthy.

The President then called upon Prof. C. N. Pierce, of Philadelphia, who read a paper, entitled "The Recuperative Power of the Tooth." The essayist accepted the doctrine that every part of the tooth, except the enamel, is alive, and subject to structural changes, and anything that will disturb the vital functions of the general system will impair the nutrition of the teeth. In other respects Prof. Pierce adhered to the old cell doctrine. The essayist believed that the arrest of caries of teeth in adult persons is due to systemic changes, and occurs usually when but little lime-salts are required by the other tissues. He, therefore, advised systemic treatment in cases of fractured roots or exposed pulps, where new formations of bone are desired. The essayist showed an enlarged plaster model of an upper central incisor which was fractured and had been united again. The fracture commenced at the lingual portion of the neck of the tooth, and ran obliquely downward and forward to about the middle of the root. The crown was held in position for six weeks by a gold cap, this being secured by cement and ligatures. The patient was about fourteen years of age, male, with a good constitution.

Dr. Wm. H. Atkinson was very glad that this subject had been brought before the society, and spoke of a similar case of the united fractures of several incisors in the mouth of Mr. Bennett, who was exhibited before the clinic a few years ago. Most of the teeth remained alive, but one of them became discolored, although the fracture had united.

The President called upon Dr. G. W. Weld, who read a very carefully prepared paper on "Implantation and Replantation." The essayist has had a great deal of experience in replanting teeth. Whenever a patient with badly decayed front teeth had presented, it had been Dr. Weld's custom to extract them, cut off the crown, screw a porcelain crown upon the root, and after this had been filled, as usual, to reinsert it. In these instances he had a normal socket and a healthy root, which usually was not kept out of the mouth longer than fifteen or twenty minutes, and he believed that if success could be expected in such cases it was preferable to the implantation of teeth in artificial sockets, when the tooth, in some instances, had been out of the mouth for months, or even years.

All the teeth and roots which the essayist had replanted had resulted in failures, remaining in the mouth from eleven months to five years, but not in a single instance had he observed a new formation of bone around any of the roots. They were always eroded and showed signs of absorption, especially of the cementum. Their final appearance was analogous to that of partially absorbed temporary teeth. If, therefore, new bone was formed around implanted teeth, it was against the usual order of things. He believed the assertion that it is so formed had not been proven. Dr. Weld, therefore, believed the pathology of Dr. Younger false, and the practice of implanting teeth in artificial sockets erroneous. He believed the firmness of implanted and replanted teeth to arise from the fibres of the pericementum penetrating the canaliculi of the alveolus, and thereby giving the tooth a kind of mechanical attachment. Both implanted and replanted teeth he regarded as foreign bodies, which nature in time will try to throw out by means of an inflammatory action, in which osteoclasts (myloplaxes) are formed; these absorb the root and produce the characteristic bay-like excavations. The essayist then spoke of living and dead tissues and cells, and also of the late Dr. Austin Flint's predictions, that many things which at present appear to be impossible, will, in the future, be accomplished, but replantation and implantation of teeth with our present methods he regarded an absolute failure.

Dr. C. Heitzmann congratulated the reader of the paper for the honesty and truthfulness with which he admitted his failures in replanting teeth. He, however, would blame Dr. Weld for not having experimented on animals, that he might demonstrate the manner in which replanted teeth become fixed in the socket. There is something far superior to any quoted authority, and that is personal experience and demonstration. The idea of Dr. Sudduth, that connective tissue-fibres grow into the empty canaliculi of the nascent corpuscles is not tenable, for it would mean about the same as to maintain that an arm of a man could grow into a button-hole. Recently, it had been asserted by a French investigator that pieces of sponge, nay of India rubber, if introduced into living tissues, would become vitalized and produce blood and blood-vessels. Dr. Heitzmann ridiculed such an idea, still more its approval by one of our leading medical papers. The manner of the fixation of dead bodies is very probably this: the surrounding tissue, owing to a persistent irritation, first swells and afterward becomes augmented, hypertrophied. From this hypertrophied tissue grow offshoots into the foreign body, that first becomes chemically altered, liquefied, or absorbed, and this is the source of blood-vessels in foreign bodies. The process is the same as the absorption of an ivory stick driven into living bone-tissue in the experiments of Billroth, performed many years ago.

Dead roots of dead teeth (and fifteen to twenty minutes out of the mouth seem to be enough to deprive teeth of their life) are foreign bodies, and are absorbed after several years, becoming loose. Dr. Weld has thus anticipated the interesting experiments of Dr. Younger, and it is highly probable that the latter gentleman, after five years, will have to acknowledge exactly the same failures that Dr. Weld has reported to-night.

Dr. F. Y. Clark did not agree with Drs. Weld and Heitzmann, for facts sometimes upset theories, and said he had replanted many teeth, some of which had been out of the mouth for three hours and more, and most of them had proved successful. They remained in the mouth from five to twenty-five years.

Dr. Younger had met with success, and this induced him to bring the matter before the profession. His theory, he acknowledged, might not be correct, but he based it upon his success. The oldest case of implantation was done seventeen months ago, and it was yet in good condition. He had replanted teeth four and five years ago. The question of the permanency of the operation could be settled in two or three years, but he was confident of success. As a disinfectant, he employs a solution of corrosive sublimate, one part of the salt to one thousand of water.

Dr. Wm. H. Atkinson thanked both essayists for their papers, and expressed satisfaction with the terminology of Prof. Pierce. Dr. Weld, he believed, had done a great deal of good, and although he did not agree with him in his conclusion, he yet thought it detestible to ridicule anyone for expressing opinions with which we do not agree. With regard to the operations in question, he would endorse Dr. Younger. Judging from the case of the lady whom he had seen at the meeting of the Odontological Society, he believed the operation would be successful.

Dr. J. F. P. Hodson moved a vote of thanks to Prof. C. N. Pierce, which was unanimously carried.

